# *Cryptococcus neoformans* Biofilm Formation and Quantification

**DOI:** 10.1002/cpz1.70133

**Published:** 2025-04-25

**Authors:** Oscar Romero, Davier Gutierrez‐Gongora, Jennifer Geddes‐McAlister

**Affiliations:** ^1^ Department of Molecular and Cellular Biology University of Guelph Guelph Ontario Canada

**Keywords:** biofilm, *Cryptococcus neoformans*, crystal violet, fungal pathogen, quantification, XTT

## Abstract

*Cryptococcus neoformans* is an opportunistic fungal pathogen that heads the Fungal Priority Pathogen List published by the World Health Organization (WHO) in 2022. This pathogen is a primary cause of death for immunocompromised individuals (e.g., those with HIV/AIDS, the elderly, immunotherapy recipients), causing approximately 118,000 deaths yearly worldwide. *C. neoformans* relies on virulence factors that include a polysaccharide capsule, melanin, extracellular enzymes, and thermotolerance to initiate and sustain host infection. Additionally, similar to other fungal pathogens (e.g., *Candida albicans*), *C. neoformans* may develop a biofilm organization linked to more persistent cryptococcal infections. Cryptococcal biofilms are highlighted in cases of cryptococcal meningitis, in which biofilm‐like structures form that are highly resistant to host immune response and to antifungal therapies. In this regard, fungal biofilm formation has become an important area of study as a means to improve our understanding of the mechanisms regulating biofilm formation and infection and to advance the discovery of antibiofilm therapeutics. To assess biofilm properties and compare across treatments, quantification and evaluation of cell viability are important. Herein, we describe a standardized method to establish a cryptococcal biofilm and quantify total biomass and cell viability. © 2025 The Author(s). *Current Protocols* published by Wiley Periodicals LLC.

**Basic Protocol 1**: Culturing and biofilm formation

**Basic Protocol 2**: Biofilm quantification

**Alternate Protocol**: Biofilm viability assay

## Introduction

In 2022, the World Health Organization published the first Fungal Priority Pathogens List, with *Cryptococcus neoformans* heading the critical priority group for research and clinical settings (WHO, 2022). This opportunistic fungal pathogen causes 118,000 deaths annually, primarily among patients with HIV/AIDS (Denning, 2024). Upon inhalation of fungal cells from the environment, *C. neoformans* may reside within the lungs, evading the host immune system, and then disseminate throughout the host. To infect and survive within a host, *C. neoformans* produces an array of virulence factors, such as the polysaccharide capsule (Zaragoza, 2019). *C. neoformans* also undergoes biofilm formation as a virulence mechanism during host invasion to support persistent infections (Aslanyan et al., 2017; Benaducci et al., 2016; Martinez & Casadevall, 2015). Biofilms are microbial communities embedded in an exopolymeric matrix that are formed to enable the microbes to survive hostile environments, resist external stressors, and maintain a favorable internal microenvironment (Flemming & Wingender, 2010). *C. neoformans* is a neurotropic pathogen that may invade the brain through hematogenous dissemination and develop biofilm‐like structures (i.e., cryptococcomas), which promote resistance to the host's immune response and to common antifungal drugs, such as azoles, polyenes, and pyrimidine analogues (Bermas & Geddes‐McAlister, 2020; Chastain et al., 2022; Velamakanni et al., 2014; Lin, 2009; Rodrigues et al., 1999). Several studies have focused on understanding how *C. neoformans* uses biofilm formation for infection as well as identifying putative antibiofilm treatment strategies (Alvarez et al., 2008; Martinez et al., 2008; Martinez & Casadevall, 2006; Moranova et al., 2009; Pettit et al., 2010). Comprehensive investigation of biofilms requires efficient quantification methods to enable the comparison of different genetic backgrounds, treatments, virulence traits, and environmental conditions. For cryptococcal biofilms in particular, however, standardized methodology to assess and quantify these fungal biofilms under diverse conditions is limited (Gutierrez‐Gongora et al., 2024, 2022; Martinez et al., 2006; Tavares et al., 2019). To meet this need, we provide a step‐by‐step biofilm quantification procedure for *C. neoformans*, developed through literature searching and our own experimental testing and optimization.

**Figure 1:**
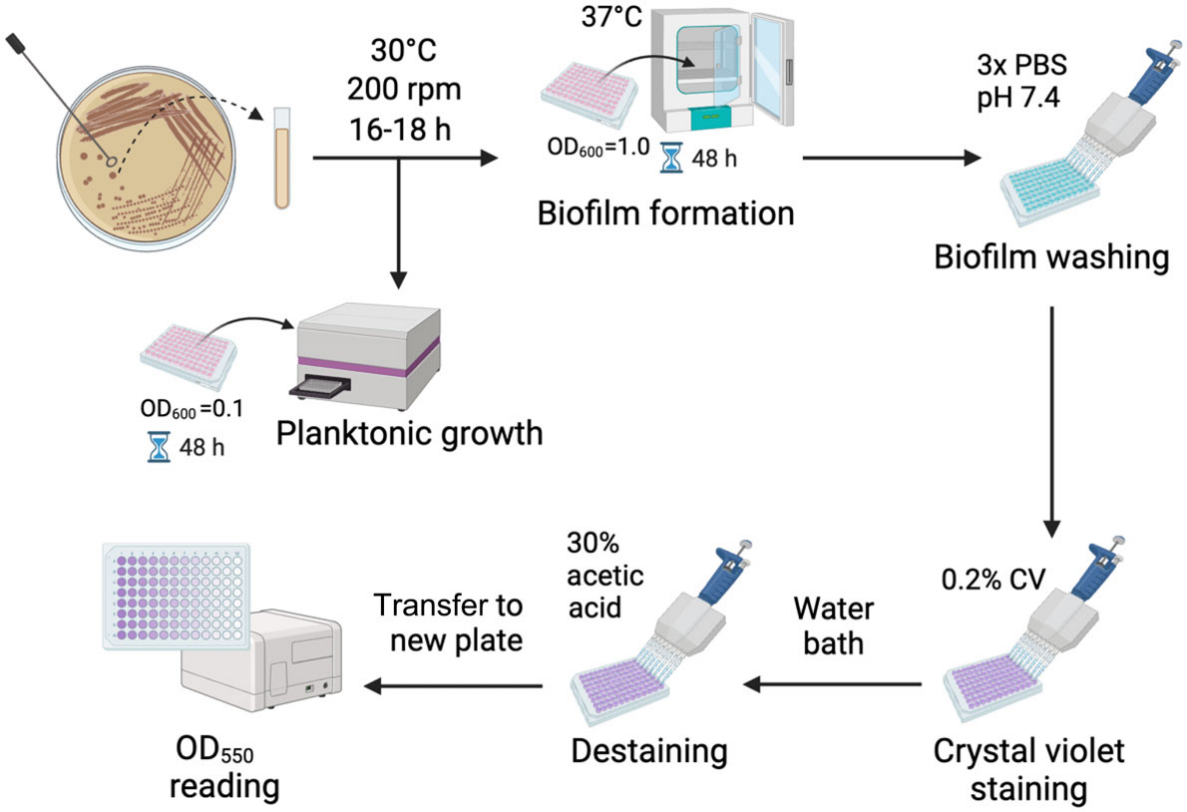
Overview of workflow for biofilm formation and quantification assay in *C. neoformans*. Isolated colonies are selected and cultured overnight at 30°C in YPD medium. The overnight culture is adjusted to OD_600_ = 1.0 (working sample) in DMEM and incubated statically at 37°C for 48 hr, alongside the working sample is adjusted to OD_600_ = 0.1 and incubated at 37°C with agitation (500 rpm) for 48 hr in a microplate reader. The biofilm culture is washed with PBS, pH 7.4, and stained with crystal violet. The excess crystal violet is removed in a water bath. Biofilms are destained with acetic acid and transferred to a new plate to measure the OD_550_.

Within this article, we focus on two methodologies for measuring cryptococcal biofilms: a crystal violet assay, which quantifies the total biomass of the biofilm, and an XTT viability assay, which enables the quantification of viable cells (Figure 1). Basic Protocol 1 describes the preparation of *C. neoformans* cells for biofilm formation, using a 96‐well plate format. Basic Protocol 2 describes the crystal violet assay for biofilm quantification. It also explains the process of measuring planktonic growth in biofilm research and how to perform this task. Finally, the Alternate Protocol describes an XTT assay for biofilm quantification. The crystal violet assay is recommended for total biofilm quantification and also is less expensive than the XTT assay, which measures only viable cells. Overall, these protocols will support the standardization of cryptococcal biofilm formation and quantification, supporting reproducibility and comparison of findings across laboratories.

*CAUTION: Cryptococcus neoformans* is a Biosafety Level 2 (BSL‐2) pathogen. All cell handling processes must be performed in a BSL‐2 laboratory, and we recommend working with the pathogen in a certified biosafety cabinet. Follow all appropriate guidelines and regulations for working with BSL‐2 microorganisms.

## Basic Protocol 1 CULTURING AND BIOFILM FORMATION

Basic Protocol 1 outlines *C. neoformans* overnight culture and biofilm formation culture set‐up. Herein, we use the wild‐type (WT) *C. neoformans* strain KN99; however, the protocol may be adapted depending on the study purposes for testing different strains and growth conditions (e.g., antibiofilm drug testing). The purpose of the experiment is to prepare biofilms from the WT and experimental (e.g., genetic mutant) strains.

### Materials

Yeast extract peptone dextrose (YPD) agar (e.g., BD Difco, cat. no. 242720)
WT *C. neoformans* strain (e.g., H99 or KN99) from glycerol stock
YPD broth (e.g., BD Difco, cat. no. 242820)
Water
1× phosphate‐buffered saline (PBS), pH 7.4 (e.g., Gibco, cat. no. 70011044)
Dulbecco's Modified Eagle Media (DMEM) with glutamine and sodium pyruvate (e.g., BioShop, cat. no. DEM001)

90‐mm petri dish (e.g., Fisher Scientific, cat. no. FB0875713A)
Biosafety cabinet
Sterile 200‐ and 1000‐µl pipet tips (e.g., Fisher Scientific, cat. no. 0540357)
37°C static incubator (e.g., Thermo Fisher Scientific, cat. no. 151030515)
20‐ml glass test tubes (e.g., Thermo Fisher Scientific, cat. no. 14‐932B)
37°C shaking incubator (e.g., Infors HT, Ecotron)
15‐ml conical tubes (e.g., Fisher Scientific, cat. no. 14955237)
Centrifuge (e.g., Eppendorf, 5430 R)
Disposable 1.5‐ml cuvettes (e.g., Fisher Scientific, cat. no. 14‐955‐127)
Spectrophotometer (e.g., Thermo Fisher Scientific, NanoDrop One/One^C^)
Flat‐bottom polystyrene 96‐well tissue culture plates (e.g., VWR, cat. no. 734‐2327)
96‐well plates (e.g., Fisher Scientific, cat. no. 21377203)
Microplate reader (e.g., Agilent, BioTek Synergy H1 Multimode Reader)

### C. neoformans culture

1 Prepare YPD agar in 90‐mm petri dishes according to the manufacturer's instructions.
  Prepare as many plates as necessary for the number of tested strains, ensuring a well‐organized and efficient process. Prepare YPD plus a specified antimicrobial for selection if working with mutant strains (e.g., YPD plus 100 µg/ml of nourseothricin). Any antimicrobial must be added after the medium is autoclaved and allowed to cool down (to ∼50°C).
2 Streak the WT *C. neoformans* strain from a glycerol stock onto YPD plate(s) using a sterile pipet tip.
  It is important to streak properly so that isolated colonies are obtained. Sterile streaking loops may also be used in place of pipet tips.
3 Incubate the cultures at 30°C in a static incubator for 48 hr or until colonies appear.
  At this stage, the cultures may be stored at 4°C until needed for the experiment. However, avoid storage for >1 month as the cultures may no longer be viable.
4 Select a single isolated colony from each culture plate using a sterile pipet tip and inoculate into 5 ml of YPD broth in 20‐ml glass test tubes.
  Use a different test tube for each colony. Setting up three or more biological replicates is recommended to obtain statistical significance.
5 Incubate overnight at 37°C in a shaking incubator at 200 rpm.

### Cell collection and preparation

6 Transfer 5 ml of each overnight culture to a 15‐ml conical tube and centrifuge for 5 min at 1500 × *g*, room temperature, to pellet the cells. Gently remove the supernatant.
7 Resuspend the cell pellet in 5 ml PBS, pH 7.4.
  Avoid using a vortex mixer as that could disturb the cells, which may affect biofilm formation.
8 Place 900 µl water in a 1‐ml cuvette. Remove 100 µl of each sample and place in the cuvette. Measure the optical density at 600 nm (OD_600_) with the spectrophotometer.
  Before adding the sample, the water can be added with the same tip to the cuvettes of different samples.
9 Use the formula C_1_V_1_ = C_2_V_2_ to calculate the volume of the cell pellet resuspended in 5 ml PBS needed to prepare a new cell suspension of OD_600_ = 1.0 (working sample) in 5 ml DMEM.
  An OD_600_ of 1.0 corresponds to ∼1.0 × 10^7^ cells/ml for C. neoformans.

### Biofilm formation culture

10 Transfer 100 µl of each working sample to individual wells in a tissue culture 96‐well plate. Fill the edge wells with water or sterile DMEM.
  Avoid using the edge wells because that may cause an edge effect, which leads to unreliable experimental results. Including technical replicates is recommended to provide more statistically reliable results. Preferably, also include a few wells containing sterile DMEM, as a blank control for the experiment. Filling the edge wells will reduce evaporation and temperature variation in the inner wells. Keep track of the sample locations in the plate.
11 Cover with the plate lid and seal with Parafilm. Incubate 48 hr at 37°C in a static incubator (at this point, the plate is ready for you to continue to Basic Protocol 2).
  Parafilm sealing also reduces water evaporation in the plate.

### Planktonic growth culture

12 Fill the inner wells of a 96‐well plate with 180 µl sterile DMEM.
13 Transfer 20 µl of each working sample to a prefilled well. Fill the edge wells with 200 µl water or sterile DMEM.
  Keep track of the sample locations in the plate, as in the biofilm plate.
14 Cover with the plate lid. Set up a 37°C growth curve at 800 rpm for 48 hr in the microplate reader. The data analysis will be performed along with the biofilm quantification results (continue to Basic Protocol 2).
  It is advisable to set up reading time points at OD_600_ in the growth curve for every 4 hr. However, taking readings every 24 hr is enough to obtain reliable results about the planktonic growth of the culture.

## Basic Protocol 2 BIOFILM QUANTIFICATION

Basic Protocol 2 outlines biofilm quantification using crystal violet to measure the total biomass, as well as how to perform the associated data analysis. The purpose of this protocol is to quantiatively compare biofilm growth between the WT and experimental (e.g., genetic mutant) strains.

### Materials

Biosafety cabinet
*C. neoformans* biofilm plate (Basic Protocol 1, step 11)
200‐ and 1000‐µl sterile pipet tips (e.g., Fisher Scientific, cat. no. 0540357)
1× phosphate‐buffered saline (PBS), pH 7.4 (e.g., Gibco, cat. no. 70011044)
0.2% (w/v) crystal violet solution (see recipe)
30% (v/v) acetic acid (see recipe)

Two water baths
Biohazardous waste container
96‐well plates, flat‐bottom, polystyrene (e.g., Fisher Scientific, cat. no. 21377203)
Microplate reader (e.g., Agilent, BioTek Synergy H1 Multimode Reader)

### Biofilm quantification

**Figure 2:**
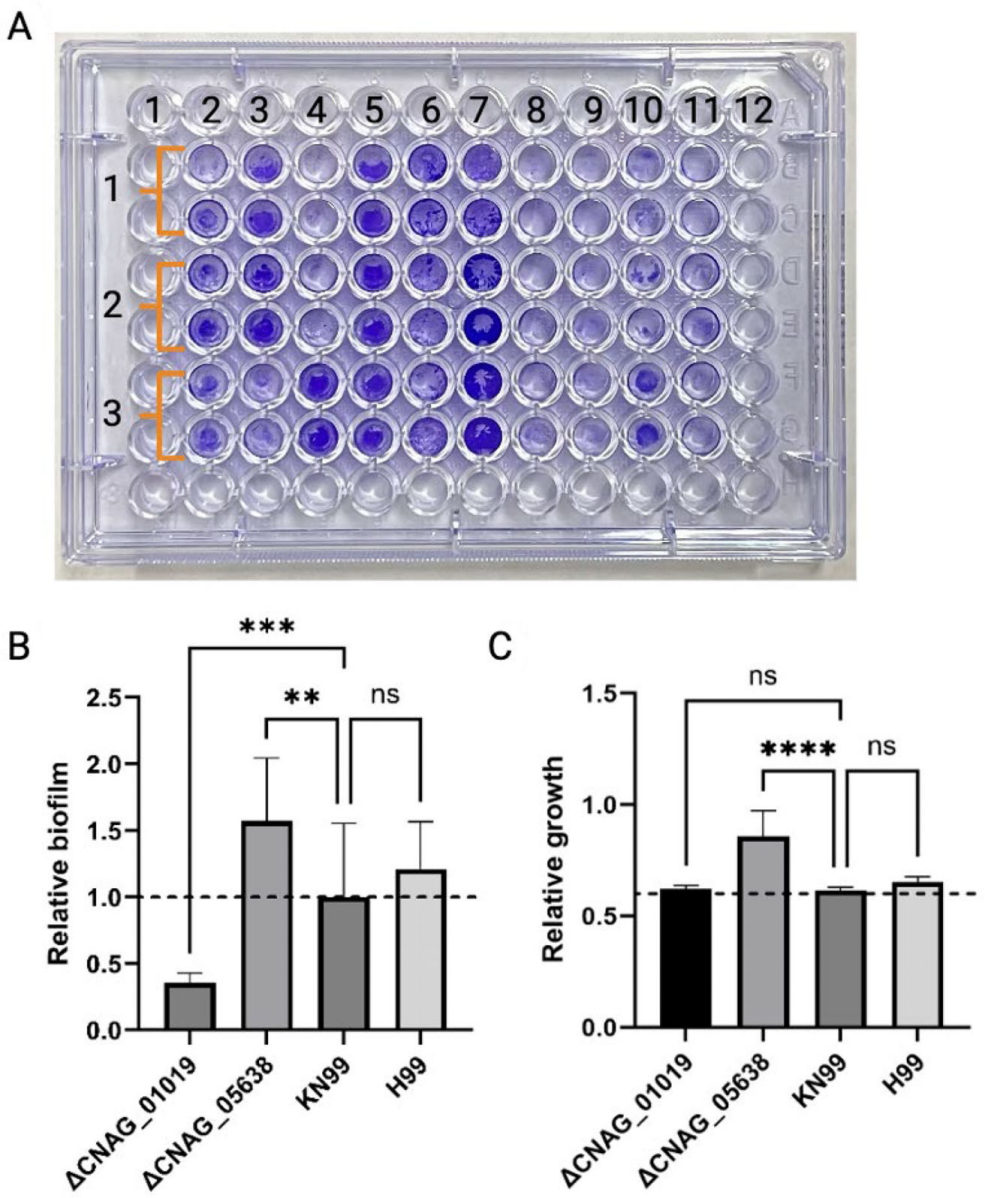
Example of expected biofilm quantification results from *C. neoformans*. (**A**) The plate is a visual representation of the biofilms after staining with crystal violet. Each column (2‐11) represents a unique *C. neoformans* strain, with strains H99 and KN99 in columns 2 and 3, respectively, while the other wells contain genetic mutants. Each strain is represented by three biological replicates (indicated by orange brackets) and two technical replicates. (**B**) Relative biofilm formation from KN99, H99, and two mutant strains (KN99 genetic background) measured with the crystal violet assay (Basic Protocol 2, step 7) after 48 hr at 37°C incubation. (**C**) Relative planktonic growth of the WT and mutant strains after 48 hr at 37°C and 800 rpm incubation (Basic Protocol 1, step 14). Three biological replicates and two technical replicates were included for each strain in both assays. Biofilms were normalized to the KN99 strain. The area under the curve was calculated from the growth curve of each strain. Statistical analysis was performed with two‐way ANOVA and Dunnett's multiple comparison test: ****p < .0001; ***p < .001; **p < .01; ns, no significance.

1 Using the biofilm plate, gently aspirate the supernatant from the wells along the side walls of each well, avoiding touching the bottom of the well. Dispose of the supernatant in a biohazardous waste container.
  C. neoformans forms biofilms on the bottom of the well. This biofilm is fragile, so it is important to avoid touching the bottom as that could disrupt the biofilm.
2 Gently wash the biofilm with 200 µl PBS slowly dispensed along the side wall of the well. Aspirate the supernatant without touching the bottom and dispose of it in the biohazardous waste container. Repeat this step three times.
  The wash step is important to remove any planktonic cells deposited on the biofilm, giving more realistic results.
3 Let the plate air dry at room temperature for 1 hr or until no liquid is visible at the bottom of the well.
4 Add 100 µl of 0.2% crystal violet solution to the wells and let stain for 10 min at room temperature.
  Do not forget to include the blank controls (i.e., wells with only DMEM) in the staining step.
  CAUTION: Crystal violet is a biohazard. Use with personal protective equipment and dispose of within designated biohazard containers.
5 Quickly submerge the plate in a water bath at room temperature for 2 s and then pour the liquid into a designated container for crystal violet residues. Repeat once. Then, submerge the plate in a second clean water bath at room temperature and discard the liquid into the same container. Let the plate air dry at room temperature for 1 hr.
  Tapping the plate with a paper towel may help remove any water remnants more efficiently. Also, having airflow (e.g., within a fume hood) may accelerate the drying process.
6 Add 200 µl of 30% acetic acid to the wells and incubate 10 min at room temperature.
  The acetic acid solubilizes the crystal violet, thereby destaining the biofilms. Gently mixing the solution in the well with a pipet may help homogenize it. Another option is to put the plate in a shaker at 500 rpm; however, the pipet method is less disruptive to the biofilms, avoiding contamination with cell debris.
7 Transfer the destaining solution to a fresh 96‐well plate, avoiding bringing along any cell debris. Read OD_550_ in the microplate reader (Fig. 2A).

### Biofilm data analysis

8 Calculate the average OD_550_ of the blank and subtract this value from each sample. Then, calculate the average OD_550_ for the WT control, and normalize each experimental sample to this value by using the formula:
$$\mathrm{normalized}\;\mathrm{value}=\left(\frac{1}{\bar{x}}\right)*x$$
  where $\bar{x}$ is the WT average and *x* is the absorbance.
9 Perform a one‐way ANOVA and a Dunnett's multiple comparison test for each experimental group against the WT control. Visualize the data in a column plot (Fig. 2B).

### Planktonic data analysis

10 Calculate the average of the blank for each desired timepoint within 48 hr and subtract this value from the value for each sample.
11 Perform an area under the curve (AUC) analysis for each sample. Then, perform a one‐way ANOVA and a Dunnett's multiple comparison test against the WT control for each experimental group using the average AUC, the standard deviation (*σ*), and the sample size (*N*). Visualize the data in a column plot. Comparison between the WT and experimental (e.g., genetic mutant) strains is performed (Fig. 2C).

## BIOFILM VIABILITY ASSAY

This alternate protocol outlines the use of an XTT kit to quantify the cryptococcal biofilm based on viable cells. XTT is a tetrazolium salt that, when reduced by metabolically active cells, converts to a soluble, orange formazan dye, enabling cell viability to be quantified by measuring the absorbance of the cell suspension at a wavelength absorbed by the dye. This approach may be appropriate when antifungal drugs are tested for disrupting biofilm structure and impacting cell viability. The purpose of this protocol is to compare biofilm viability between WT and experimental (e.g., genetic mutant) strains.

### Additional Materials (also see Basic Protocol 2)

XTT (2,3‐bis‐(2‐methoxy‐4‐nitro‐5‐sulfophenyl)‐2*H*‐tetrazolium‐5‐carboxanilide) cell proliferation kit (e.g., Roche, cat. no. 11465015001); prepare working solution as per the manufacturer's instructions before beginning the protocol

1 Follow Basic Protocol 2, steps 1 and 2 (biofilm wash).

### Biofilm viability measurement

2 Add 100 µl DMEM to the wells.
  Be careful when adding the medium, avoiding biofilm disruption.
3 Add 50 µl XTT working solution.
  You must include a blank control (i.e., sterile medium) with the XTT.
4 Incubate the plate for 4 hr at 37°C in the static incubator.
5 Measure OD_450_ and OD_660_ with the microplate reader.
  The OD_450_ is used to measure the formazan salt present in the sample, whereas the OD_660n_ is used as a reference to eliminate the background signal caused by cell debris.
6 Use the formula below to calculate the specific absorbance for the formazan salt (where *A*_r_ = real absorbance):
*A*_r_ = [*A*_450_(test) – *A*_450_(blank)] – [*A*_660_(test) – *A*_660_(blank)]
7 Follow Basic Protocol 2, steps 8 and 9, for data analysis.

## REAGENTS AND SOLUTIONS

### Crystal violet, 0.2% (w/v)

0.2 g crystal violet powder (Sigma‐Aldrich, cat. no. C6158)
20 ml methanol (Supelco, cat. no. 103726; 20% [v/v] final)
Dilute to 100 ml with Milli‐Q‐purified water
Store up to 1 month at room temperature in an amber container.

### Acetic acid, 30% (v/v)

300 ml acetic acid (Fisher Scientific, cat. no. A507‐P500)
700 ml Milli‐Q‐purified water
Store up to 1 month at room temperature.

## Commentary

### Critical Parameters and Troubleshooting

During *C. neoformans* cell collection and preparation, it is important to avoid disturbing the cells as this could affect biofilm formation. Also, once the cells are collected, it is recommended that the protocol be continued until the biofilm formation cultures are finished; avoid storage of the cells for future use. In the biofilm quantification steps, be careful when aspirating the supernatant, because this may damage the biofilms, potentially introducing variability into the results. Leave the crystal violet to stain for the indicated time or longer, as this step also fixes the biofilm in the plate and allows the preservation of the biomass. Similarly, allow the destaining step to continue to completion, and only transfer the solution when homogeneity is obvious. Importantly, always include a blank control (i.e., medium‐only well) across all steps in this protocol. Table 1 presents a guide for troubleshooting in the biofilm formation assay.

**Table 1:** Troubleshooting Guide for Biofilm Formation Measurement

Problem	Possible cause	Solution
Contamination	There is a common contaminant microorganism in the working space	Use plates containing YPD plus chloramphenicol (100 µg/ml).
Poor biofilm formation	The medium used to induce the biofilm formation is not optimal	Try using other types of media, such as RPMI‐1640, a minimal medium, or artificial cerebrospinal fluid (aCSF).
Biofilm loss during wash steps	Disruption and aspiration of the biofilm	Reducing the number of wash steps may give more consistency in the results. Also, other procedural changes, such as using electronic pipets, may help (e.g., Eppendorf 12‐channel Xplorer plus).

### Understanding Results

Differences in biofilm formation can be described through the implementation of this protocol; such differences are visible after the biofilms are stained (Fig. 2A). Visible similarity between the technical replicates of one biological replicate indicates correct execution of the protocol. The quantified biofilm should be normalized to the control; in this case, *C. neoformans* KN99 was used to normalize the biofilms of the other tested strains (Fig. 2B). Here, we included two mutant strains (i.e., CNAG_01019 and CNAG_05638) with a KN99 genetic background to show differences in the biofilm formation. CNAG_01019 encodes superoxide dismutase 1 (Sod1), previously suggested to be involved in cryptococcal biofilm formation (Santi et al., 2014). This enzyme is essential for oxidative stress response, a critical process for biofilm defenses. For instance, deletion of *sod1* causes a >50% reduction in biofilm compared to that for WT KN99. On the other hand, CNAG_05638 encodes a hypothetical protein that was also found to be upregulated during biofilm formation (Santi et al., 2014). However, the deletion of this gene causes an increase in biofilm production. Notably, biofilm formation may be significantly affected by the growth fitness of the strain, and it is therefore important to include the assessment of planktonic growth within the assay. In our results presented in Figure 2C, we demonstrate a correlation between increased biofilm and enhanced fungal growth for the CNAG_05638 gene deletion strain, whereas the *sod1* mutant showed no significant difference from the control for planktonic cell growth.

### Time Considerations

Initial culturing of *C. neoformans* on YPD plates starting from glycerol stock requires at least 48 hr. However, depending on the strain, this time may vary. Overnight cultures of the selected colonies require an incubation of 16‐18 hr in liquid medium, with the time again varying depending upon strain growth. The procedure for cell collection and preparation, biofilm formation, and planktonic culturing requires 3‐5 hr, depending on the number of strains and conditions being tested. These cultures are then incubated for 48 hr. Finally, biofilm quantification takes ∼2 hr for the crystal violet protocol and ∼5 hr for the XTT viability protocol.

### Author Contributions

**Oscar Romero**: Conceptualization; data curation; formal analysis; investigation; methodology; validation; visualization; writing–original draft; writing–review and editing. **Davier Gutierrez‐Gongora**: Conceptualization; methodology; writing–review and editing. **Jennifer Geddes‐McAlister**: Conceptualization; funding acquisition; methodology; project administration; resources; supervision; validation; visualization; writing–original draft; writing–review and editing.

### Conflict of Interest

The authors declare no conflicts of interest.

## Data Availability

The data that support the findings of this study are available from the corresponding author upon reasonable request.
